# Peri-implant bone behavior after single drilling technique versus undersized drilling technique of immediately loaded implant in posterior maxilla: a one-year prospective study

**DOI:** 10.1186/s12903-025-06360-0

**Published:** 2025-06-21

**Authors:** Mohamed Ahmed Galal Sadek, Mohamed Zaghlool Amer, Nesma El-Gohary, Heba Abo-Elfetouh Elsheikh

**Affiliations:** 1 Department of Oral and Maxillofacial Surgery, Faculty of Dentistry, Mansoura University, Mansoura, Egypt; 2 Department of Fixed Prosthodontics, Faculty of Dentistry, Mansoura University, Mansoura, Egypt

**Keywords:** Implant stability, Immediate loading, Undersized drilling technique, Single drilling technique, Posterior maxilla

## Abstract

**Background:**

Implant placement in the posterior maxilla is challenging, so modifications of the surgical techniques were introduced to overcome these challenges. The undersized drilling technique uses a final drill smaller than the diameter of the implant. The single drilling technique is a simplified method where the osteotomy is made using a single drill without sequential widening. This study was directed to evaluate the peri-implant bone behavior of the undersized drilling technique versus the single drilling technique of immediately loaded dental implants inserted in the posterior maxilla.

**Patients and methods:**

32 patients were selected for prosthetic replacement of a missing maxillary posterior single tooth by an immediately loaded dental implant and divided randomly into two equal groups. In Group I: 16 patients received 16 implants using the undersized drilling technique, while in Group II: 16 patients received 16 implants using the single drilling technique. Insertion torque, implant stability, modified sulcus bleeding index (mBI), peri-implant probing depth, bone density, and marginal bone height were evaluated for both groups. Statistical analysis was made for clinical and radiographic data.

**Results:**

32 implants were inserted in the posterior maxilla. During a 12-month follow-up, every dental implant was successful with no complications. Both techniques showed high insertion torque (≥ 35 Ncm) and primary stability (> 70 ISQ) with no significant difference between the two groups (*P* > 0.05). Also, there were no significant differences between the study groups regarding peri-implant soft tissue health, bone density, and marginal bone loss (*P* > 0.05).

**Conclusion:**

Both techniques revealed comparable, promising clinical and radiographic outcomes over a 12-month post-loading follow-up period when the immediate loading protocol was used in the posterior maxilla, where bone density is poor, but preparing the implant bed using the single drilling technique offers several merits for both the patient and clinician. In addition to avoiding excessive heat generation, mechanical damage, and high frictional forces during drilling procedures, surgical operations, and surgical site exposure take less time.

**Trial registration:**

Clinical-Trials.gov PRS (https://register.clinicaltrials.gov) had this study registered under the identifier number. NCT06770231 on 01/01/2025.

## Background

Replacing single or multiple missing teeth with dental implants is a reliable treatment option that aims to restore the patient’s function, comfort, and aesthetics [1]. The maxillary posterior region is challenging for implant placement as it has inadequate inter-arch space, decreased ridge width and height, and poor bone quality [2].

Primary implant stability is essentially a crucial factor in determining the implant’s long-term success, as it creates the foundation for secondary implant stability. A biological network between the implant and the surrounding bone does not exist once the implant is placed. Secondary or biological stability is the outcome of osseointegration, which follows bone formation and remodeling [3]. Numerous factors, such as the surgical technique, implant design, and patient-specific characteristics like bone shape and quality, affect primary stability [4]. The clinician uses the primary stability to assist in the choice of when to load the implant. It is advised to use the conventional loading protocol if the primary stability is questionable. Conversely, immediate implant loading is possible if the primary stability is high [3].

Immediate loading of dental implants in the posterior maxilla is a difficult process, but it could be achieved by reaching high implant primary stability and insertion torque values to decrease implant micro-motion that would prevent osseointegration [5]. Different surgical technique modifications were introduced to overcome such challenges in the posterior maxilla to improve the primary stability and insertion torque to allow successful implant placement [6].

One of these surgical modifications is called the undersized drilling technique, which is defined as using a final drill smaller than the diameter of the implant. Comparing the size of the final drill to the implant size is known as the undersizing percentage. It was shown that 10% undersizing is better than 25% undersizing [7]. Improving primary stability can be achieved most easily and effectively by underpreparing the implant site [7]. However, the main drawback of this technique is that overcompression of the crestal bone can lead to necrosis. To avoid this complication, the clinician must accurately assess the bone density [8].

The undersized drilling technique increases bone density, which in turn increases primary stability and insertion torque in type IV, low-quality bone, and occasionally even type III bone. This technique appears to offer little advantage in situations where the bone exhibits acceptable quality, such as type I and II, and it may also impair the bone’s vascularization [9].

A progressive drilling sequence has been regarded as a key component of successful implant placement. Nevertheless, it takes time to use multiple drills at various stages, which has led to several drawbacks, including increased infection risk and patient discomfort [10]. If reducing the number of steps in the drilling process does not negatively affect success, then it would be meaningful. It has been stated that simplifying the drilling process has produced satisfactory outcomes; nevertheless, the majority of these studies used animals [11, 12].

The single drilling technique is a simplified method that eliminates the need for many drills by utilizing a single drill that precisely matches the implant’s diameter [13]. The idea of drilling with a single drill has been supported by multiple studies, which noted that the temperatures during drilling do not rise to levels that are detrimental to the bone [14, 15]. Furthermore, a different study found that the single drilling approach does not affect the biomechanical and/or biological response of the surrounding tissues during implant site preparation, making it a safe technique for implant bed osteotomy [16].

A new single drilling system with a hollow design was established. This design was introduced to reduce the time of the surgery and minimize bone removal during osteotomy preparation, as it prevents repeated drilling that might cause heat stress to bone tissue and extended exposure of the operative site. Also, it leads to a better healing response and allows the harvesting of an autogenous bone grafting material, which can be used for further surgical purposes [17].

The aim of the study was directed to evaluate the peri-implant bone behavior of the undersized drilling technique versus the single drilling technique of immediately loaded dental implants inserted in the posterior maxilla. The study’s null hypothesis stated that there was no significant difference between the undersized drilling technique and the single drilling technique regarding insertion torque, implant stability, modified sulcus bleeding index, peri-implant probing depth, bone density, and marginal bone loss (MBL).

## Patients and methods

### Patient selection

32 patients were chosen to have dental implants to replace their lost maxillary posterior tooth/teeth. Every patient was chosen from the Mansoura University, Faculty of Dentistry, Oral and Maxillofacial Surgery Department’s outpatient clinic.

Before the proposed surgery, all involved patients provided written informed consent after being fully apprised of the potential risks and advantages of the procedure. Under protocol number A03012024OS, the current study was authorized by the Institutional Review Board (IRB) of the Faculty of Dentistry, Mansoura University, Mansoura, Egypt, following the seventh revision of the Helsinki Declaration in 2013. On 01/01/2025, Clinical-Trials.gov PRS (https://register.clinicaltrials.gov) had this study registered under the identifier number. NCT06770231.

### Criteria for patient selection

**Table:** 

Inclusion criteria	Exclusion Criteria
1. Patients who have lost one or more of their maxillary posterior teeth.	1. Root apex at the planned implant site.
2. Patients age ≥ 18 years.	2. Drug or alcohol abuse.
3. Bone height was 10 mm or more.	3. Patients with uncontrolled systemic diseases that contraindicate the implant surgery.
4. Patients who were willing to attend the follow-up appointments and maintain proper dental hygiene.	4. Pregnant women.
5. Sufficient inter-arch space and mesio-distal width	5. Smokers.
6. Patients free of para-functional habits including bruxism and clenching.	

### Sample size calculation

Calculating the sample size was dependent on the implant stability under various surgical drilling techniques retrieved from a previous study (Elsheikh et al., 2022) [18]. Using G*Power program version 3.1.9.7 to calculate sample size based on an effect size of 1.20, using a 2-tailed test, α error = 0.05, and power = 90.0%, the total calculated sample size was 16 in each group.

### Study design

The CONSORT criteria for conducting clinical trials were followed in this prospective randomized clinical trial (CONSORT Flowchart). Two equal groups of patients were randomly assigned to this trial:



#### Group I

16 immediately loaded dental implants were inserted in 16 patients using the undersized drilling technique.

#### Group II

16 immediately loaded dental implants were inserted in 16 patients using the single drilling technique.

### Randomization

A department senior resident who was not involved in the trial and was unaware of the associated treatment plan assigned each missing tooth to the study. A computer-generated randomization list (SPSS v27.0) was used to ensure uniform randomization. Each group was composed of 16 implants. The groups were distributed as follows: Group I (Undersized drilling technique) and Group II (Single drilling technique).

### Preoperative measures

At the intended implant site, preoperative cone beam computed tomography (CBCT) was performed using OnDemand 3D (Cybermed Co., Seoul, Korea) to assess the buccolingual width and residual bone height of the lost tooth.

**Fig. 1:**
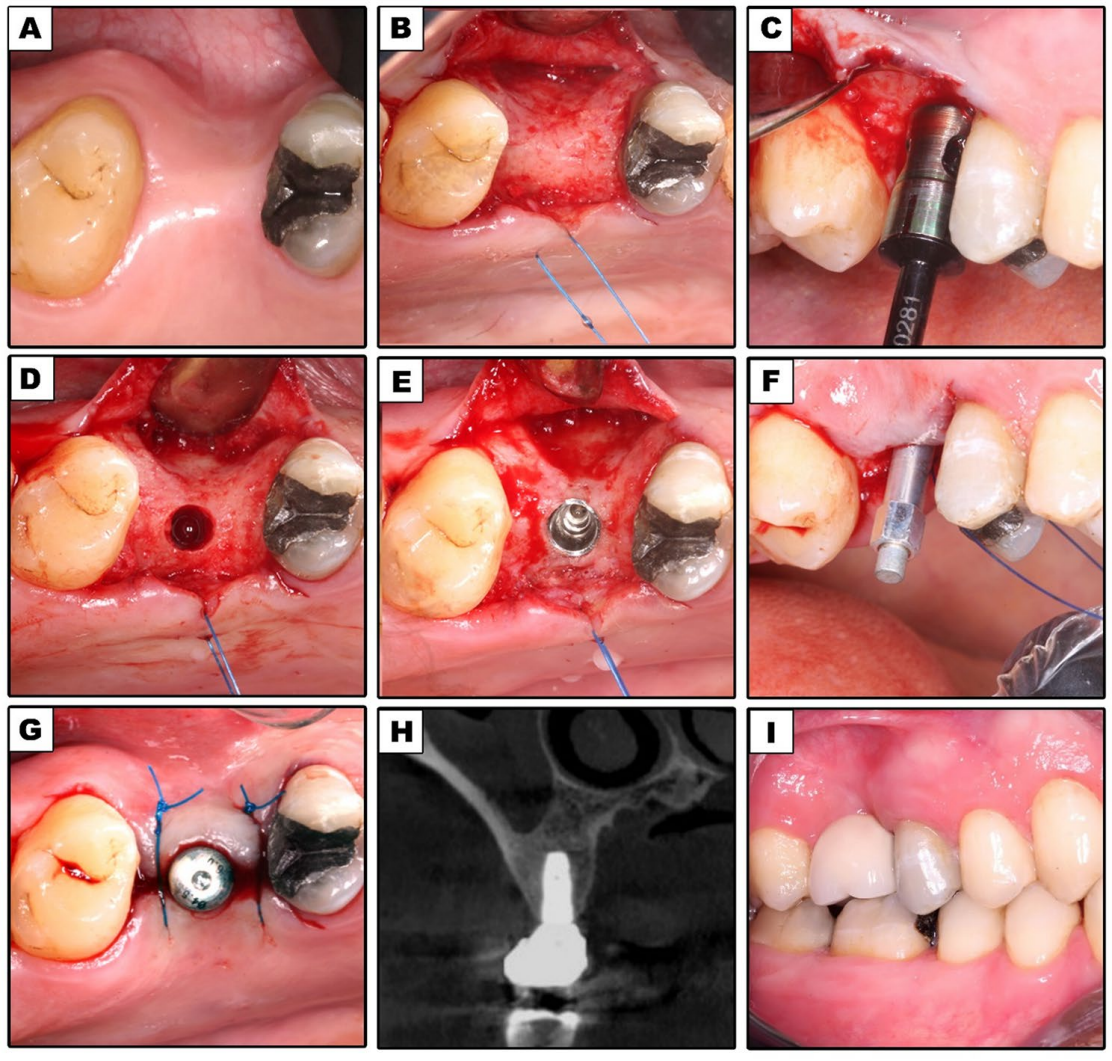
Undersized Group. (**A**) Preoperative intraoral occlusal view of planned implant site; (**B**) Elevation of a full-thickness mucoperiosteal flap; (**C**) Placement of final drill in osteotomy site; (**D**) Final osteotomy site before implant placement; (**E**) Dental implant placed in osteotomy site; (**F**) Smart peg attached to dental implant; (**G**) Healing abutment attached to implant; (**H**) A cross-sectional CBCT image immediately postoperative; (**I**) Final restoration in place

**Fig. 2:**
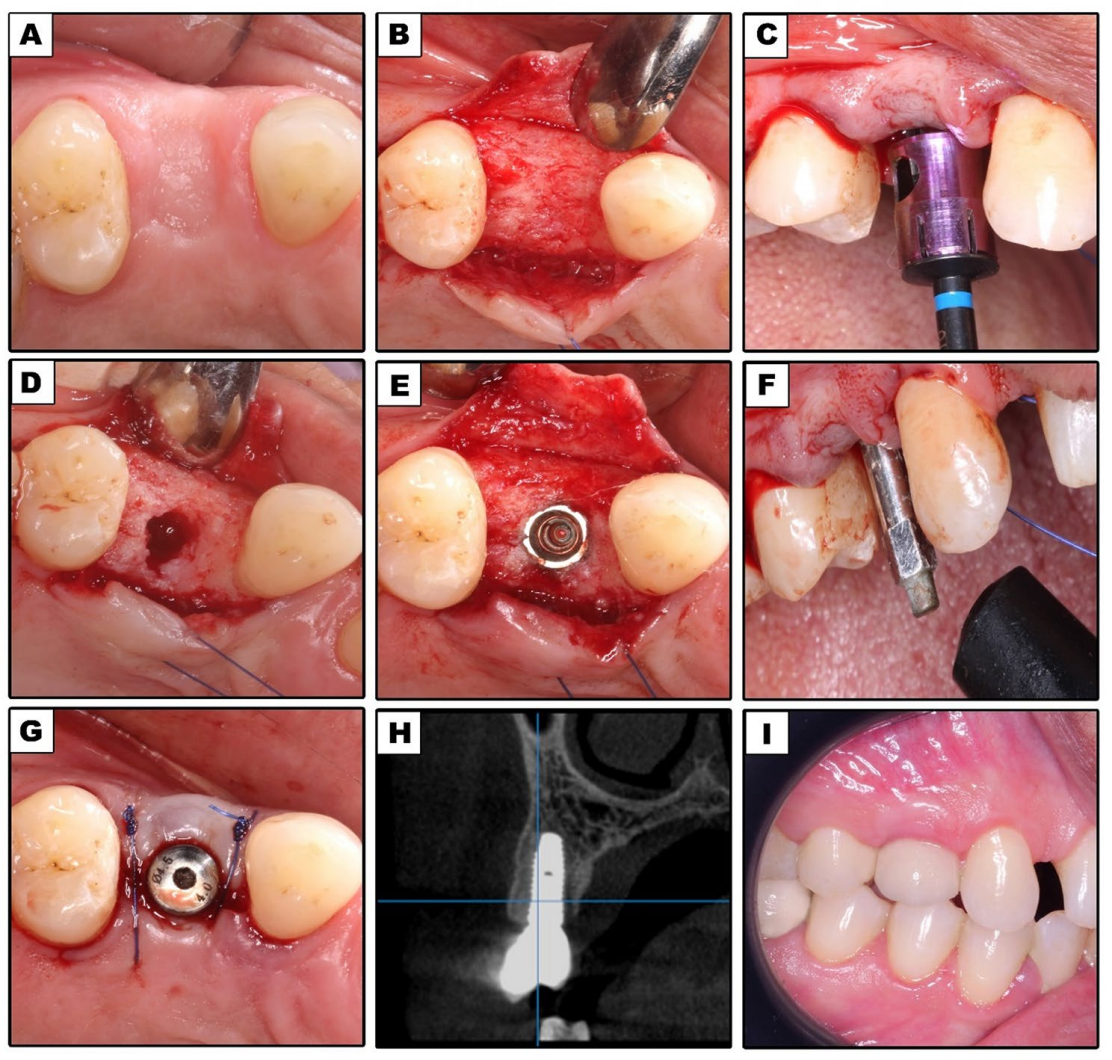
Single Drill Group. (**A**) Preoperative intraoral occlusal view of planned implant site; (**B**) Elevation of a full-thickness mucoperiosteal flap; (**C**) Placement of single drill in osteotomy site; (**D**) Final osteotomy site before implant placement; (**E**) Dental implant placed in osteotomy site; (**F**) Smart peg attached to dental implant; (**G**) Healing abutment attached to implant; (**H**) A cross-sectional CBCT image immediately postoperative; (**I**) Final restoration in place

Amoxicillin and Clavulanic acid 1gm tablets (Augmentin, Medical Union Pharmaceuticals, Egypt for GSK) were administered as part of a prophylactic antibiotic regimen two hours before surgery. A one-minute rinse with Chlorohexidine mouthwash (Hexitol mouthwash 100 ml by (ADCO) – A.R.E.) was prescribed before surgery. As a baseline, preoperative intraoral photographs had been collected (Figs. 1A and 2A).

### Surgical procedures

Articane HCL 4% -1:100000 adrenaline was used for buccal and palatal infiltrations for all surgical operations, then the crestal bone was exposed by reflecting a full thickness paracrestal mucoperiosteal flap (Figs. 1B and 2B).

In Group I, the final drill was skipped, but otherwise the implant site preparation followed the manufacturer’s instructions. The drilling speed was 800–1200 rpm according to bone density (Fig. 1C).

**Fig. 3:**
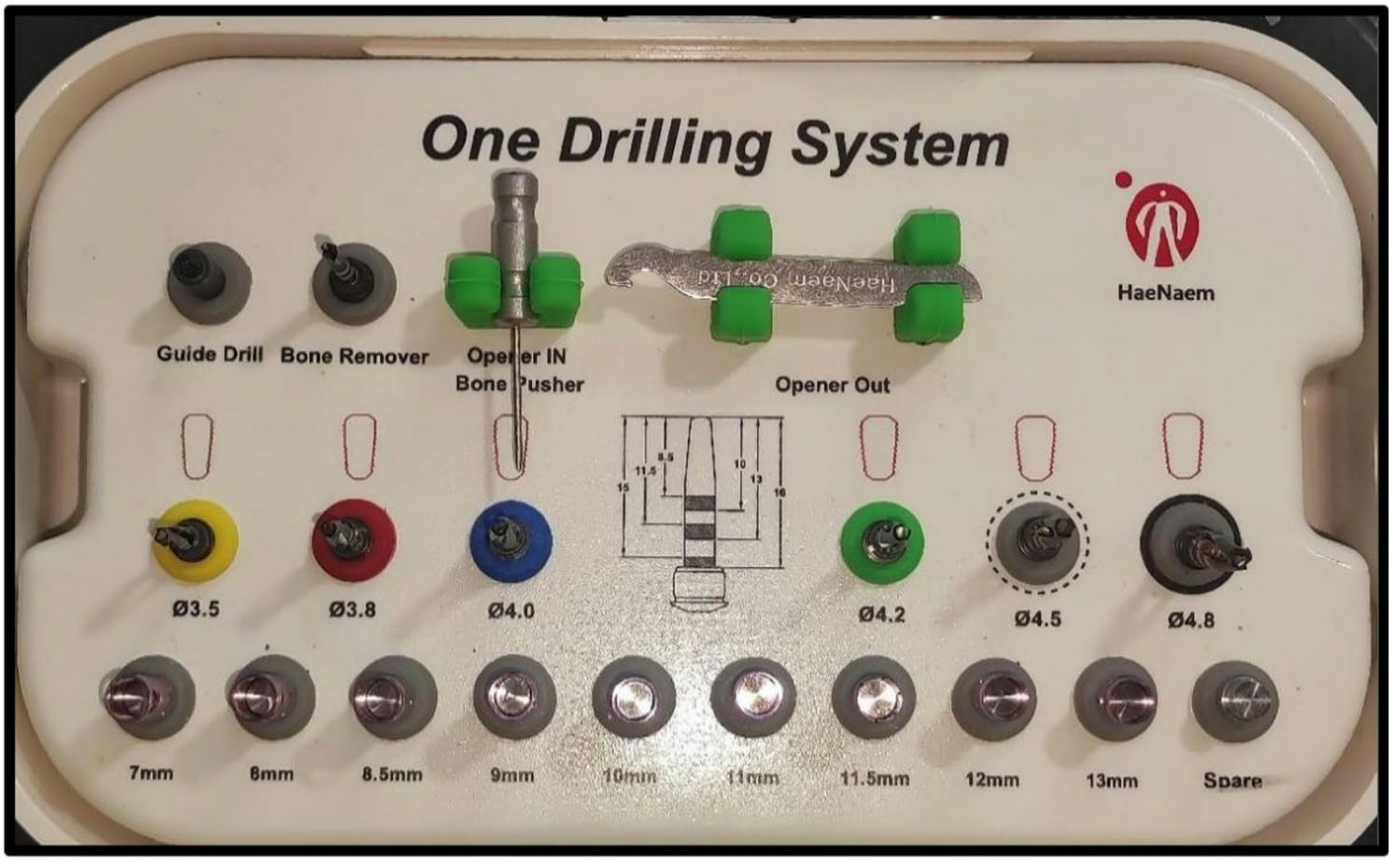
One Drill System Kit

In Group II, the new, specially made drills (One Drill System Kit, HaeNaem Co., Ltd, Korea) were used to prepare the implant bed. (Fig. 3) A guiding drill of 3.5 mm in length at a speed of 800 rpm was used first, followed by a drill with the same diameter as the chosen implant size used along the osteotomy site (Fig. 2C).

For both Groups, the sterile implant package (Neobiotech^®^ system, IS II active, Seoul, Korea) was opened following the completion of the osteotomy (Figs. 1D and 2D), and the implant was placed 1 mm below the alveolar crest using steady, mild finger pressure (Figs. 1E and 2E). A manual ratchet was used to record the insertion torque. The flap was repositioned and sutured using simple interrupted 4/0 prolene sutures after placement of the healing abutment (Figs. 1G and 2G).

### Postoperative care and instructions

On the first day of operation, the patient was told to perform intermittent cold fomentations every 20 min. The patient was counseled to continue using mouthwash containing chlorohexidine in order to maintain proper oral hygiene.

For five days following surgery, all patients were kept on one gram of a combination tablet (amoxicillin + clavulanic acid) twice a day. For the following three days, they were also given a 600 mg ibuprofen tablet (Kahira Pharm. & Chem. Ind. Co.) as a non-steroidal anti-inflammatory drug.

### Prosthetic rehabilitation

Immediately after surgery, all implants were loaded within 48 to 72 h. The healing abutment was unscrewed and replaced with an impression post, and an impression was made utilizing the open tray technique. After examining the margins and occlusion, the final restoration that was made from metal-ceramic material was cemented using temporary cement. (Figs. 1I and 2I).

### Evaluation

#### A. Clinical evaluation

All included patients were assessed at implant loading, 3, 6, and 12 months post-loading regarding the following clinical parameters.

1. Insertion torque:

During implant insertion, the insertion torque was recorded.

2. Implant Stability:

Implant stability was assessed at implant placement, 3, 6, and 12 months after surgery. For resonance frequency analysis (RFA), an Osstell Mentor device was used. Measurements were made in four different directions at 90-degree to get the RFA value. The outcomes were averaged for every implant and reported as the implant stability quotient (ISQ) [19] (Figs. 1F and 2F).

3. **Modified Sulcus Bleeding Index (mBI)**:

3, 6, and 12 months following crown installation, the modified sulcus bleeding index (mBI) was assessed. Clinical signs of peri-implant mucosal inflammation were scored using the mBI criteria [20].

4. **Peri-implant Probing Depth:**

At loading and 3, 6, and 12 months following surgery, the peri-implant probing depth was measured. Along the vertical axis of the implant, the probe was positioned buccally, mesially, palatally, and distally. The mean value was determined after all measurements were made using the nearest 0.5 mm approximation [21].

#### B. Radiographic evaluation

CBCT was performed immediately at the time of implant loading (Figs. 1H and 2H), after 6 and 12 months with identical parameters (89 kVp and 8 mA,).

1. **Bone Density Recordings**:

Using the Hounsfield unit, relative bone density measurements were obtained from the cross-sectional view. It was recorded preoperatively at the planned implant site, immediate postoperatively, 6 and 12 months after surgery. Measurements were made parallel to the implant body, 1 mm distant. Three measurements were taken and averaged from the buccal side at the apical, middle, and coronal thirds of the implant body. The palatal side underwent the same procedure [22].

2. **Marginal Bone Loss:**

By modifying the cross-sectional and panoramic long axis to bisect the implant’s center bucco-palatally, the dental implant can be used as a reference to assess MBL. A line was drawn directly parallel to the implant in the cross-sectional view. It began at the crestal bone buccally and palatally and finished at the implant’s apical level. At loading (baseline), 6 months, and 12 months following loading, the marginal bone height was assessed in millimeters. MBL was measured by subtracting the values of 6 and 12 months from the baseline value [23].

### Statistical analysis

Data management and statistical analysis were performed using the Statistical Package for Social Sciences (SPSS v27.0). Numerical data were summarized using means, standard deviations, and ranges. Data were explored for normality using the Kolmogrov-Smirnov test and the Shapiro-Wilk test. Categorical data were summarized as percentages and compared by the Fisher exact test. Comparisons between the 2 groups were done using the independent t-test. Paired t-test was done to assess the difference between two repeated measures as well as repeated measure ANOVA was used for 3-time intervals assessment. All p-values are two-sided. P-values ≤ 0.05 were considered significant.

## Results

32 dental implants were used to replace the lost posterior maxillary teeth (premolars and molars) in a total of 32 patients, 26 of whom were female and 6 of whom were male. The patients’ ages ranged from 25 to 48 years. (Table 1). All of the implants underwent successful osseointegration during the 12-month postoperative follow-up period. The distribution of replaced teeth and implant characteristics are shown in Table 2.

**Table 1:** Demographic data (Sex and Age)

Demographic data		Group I *n* = 16	Group II *n* = 16	Test of significance
**Gender**	**Female**	12 (75%)	14 (87.5%)	*P* = 0.65
	**Male**	4 (25%)	2 (12.5%)	
**Age**	**Mean ± SD**	37.9 ± 4.2	40.3 ± 5.3	t = 1.42 *P* = 0.16
	**Range**	25–45	28–48	

SD: standard deviation, *P* < 0.05 is statistically significant, analysis was done by fisher exact test for gender and independent t test for age

**Table 2:** Replaced tooth, side distribution, implant length and implant diameter

Assessment parameter		Group I *n* = 16	Group II *n* = 16	*P* value
**Tooth replaced**	**Upper 4**	6 (37.5%)	5 (31.25%)	1
	**Upper 6**	10 (62.5%)	11 (68.75%)	
**Side distribution**	**Upper left**	4	6	0.70
	**Upper right**	12	10	
**Implant length**	**10 mm**	2 (12.5%)	2 (12.5%)	1
	**11.5 mm**	14 (87.5%)	14 (87.5%)	
**Implant diameter**	**4.5 mm**	16 (100%)	16 (100%)	1

*P* < 0.05 is statistically significant, analysis was done by fisher exact test

### A. Clinical evaluation

1. **Insertion Torque**:

There was no statistically significant difference in insertion torque value between the two groups immediately after insertion of the implant (*P* = 0.49) (Table 3).

**Table 3:** **Insertion torque:**

Assessment parameter			
	Group I *n* = 16	Group II *n* = 16	Test of significance
	Mean ± SD	Mean ± SD	
**Insertion Torque**	35 ± 2.58	35.62 ± 2.5	t = 0.69 *P* = 0.49

SD: standard deviation, *P* ≤ 0.05 is statistically significant, analysis was done by independent t test

2. **Implant Stability**:

There was no statistically significant difference regarding ISQ values between the two groups immediately postoperative and 3, 6, and 12 months after insertion of the implant (*P* > 0.05) (Table 4).

**Table 4:** Implant stability

Time of assessment			
	Group I *n* = 16	Group II *n* = 16	Test of significance
	Mean ± SD	Mean ± SD	
**Immediate postoperative**	77.31 ± 3.92	76.25 ± 5.76	t = 0.60 *P* = 0.54
**3 months**	74.37 ± 3.2	73.18 ± 3.1	t = 1.06 *P* = 0.29
**6 months**	75.81 ± 4.06	74.93 ± 4.07	t = 0.61 *P* = 0.54
**12 months**	76.12 ± 5.92	75.5 ± 4.53	t = 0.33 *P* = 0.74

SD: standard deviation, *P* ≤ 0.05 is statistically significant, analysis was done by independent t test

3. **Modified Sulcus Bleeding Index(mBI):**

There was no statistically significant difference regarding mBI between the two groups after 3, 6, and 12 months after insertion of the implant (*P* > 0.05) (Table 5).

**Table 5:** Modified sulcus bleeding index (mBI)

Time of assessment	Group I *n* = 16	Group II *n* = 16	Test of significance
	Mean ± SD	Mean ± SD	
**3 Months**	0.15 ± 0.11	0.2 ± 0.18	t = 0.94 *P* = 0.35
**6 Months**	0.22 ± 0.34	0.08 ± 0.11	t = 1.56 *P* = 0.12
**12 months**	0.1 ± 0.11	0.18 ± 0.33	t = 0.92 *P* = 0.36
**Repeated Measure ANOVA**	*P* = 0.12	*P* = 0.26	

SD: standard deviation, *P* ≤ 0.05 is statistically significant, analysis was done by independent t test. Comparing. changes overtime in each group by repeated measure ANOVA

4. **Peri-implant Probing Depth:**

There was no statistically significant difference regarding peri-implant probing depth between the two groups immediately after loading and 3, 6, and 12 months after insertion of the implant (*P* > 0.05) (Table 6).

**Table 6:** Peri-implant probing depth

Time of assessment	Group I *n* = 16	Group II *n* = 16	Test of significance
	Mean ± SD	Mean ± SD	
**At Loading**	3.67 ± 0.33	3.47 ± 0.42	t = 1.49 *P* = 0.14
**3 Months**	2.97 ± 0.36	2.64 ± 0.58	t = 1.93 *P* = 0.06
**6 Months**	2.48 ± 0.36	2.39 ± 0.37	t = 0.69 *P* = 0.49
**12 months**	2.35 ± 0.36	2.2 ± 0.28	t = 1.31 *P* = 0.19
**Repeated Measure ANOVA**	*P* < 0.001 *	*P* < 0.001*	

SD: standard deviation, *P* ≤ 0.05 is statistically significant, analysis was done by independent t test. Comparing. changes overtime in each group by repeated measure ANOVA. *: statistically significant

### B. **Radiographic evaluation**

1. **Bone Density:**

Regarding buccal and palatal bone density, there was no statistically significant difference between the two groups preoperatively, immediately postoperative, 6, and 12 months after implant insertion (*P* > 0.05) (Table 7).

**Table 7:** Buccal and palatal bone density

Time / Side of assessment	Group I *n* = 16	Group II *n* = 16	Test of significance
	Mean ± SD	Mean ± SD	
**Buccal bone density**			
**Preoperative**	300.95 ± 136.5	323.72 ± 224.26	t = 0.34 *P* = 0.73
**Immediate postoperative**	850.98 ± 255.47	815.71 ± 254.09	t = 0.39 *P* = 0.69
**6 months**	1095.54 ± 206.58	1106.97 ± 230.72	t = 0.14 *P* = 0.88
**12 months**	1107.26 ± 209.87	1114.66 ± 243.32	t = 0.09 *P* = 0.92
**Repeated Measure ANOVA**	*P* < 0.001*	*P* < 0.001*	
**Palatal bone density**			
**Preoperative**	284.77 ± 122.4	317.46 ± 169	t = 0.62 *P* = 0.53
**Immediate postoperative**	580.82 ± 213.56	720.11 ± 230.99	t = 1.77 *P* = 0.08
**6 months**	802.97 ± 361.59	780.51 ± 282.99	t = 0.21 *P* = 0.84
**12 months**	868.68 ± 559.54	864.12 ± 309.29	t = 0.03 *P* = 0.97
**Repeated Measure ANOVA**	*P* < 0.001*	*P* < 0.001*	

SD: standard deviation, *P* ≤ 0.05 is statistically significant, analysis was done by independent t test. Comparing. changes overtime in each group by repeated measure ANOVA. *: statistically significant

2. **Marginal Bone Loss:**

Regarding buccal and palatal marginal bone loss, there was no statistically significant difference between the two groups after 6, and 12 months of implant insertion (*P* > 0.05) (Table 8).

**Table 8:** Marginal bone loss (MBL)

Time / Side of assessment	Group I *n* = 16	Group II *n* = 16	Test of significance
	Mean ± SD	Mean ± SD	
**Buccal**			
**6 months**	0.34 ± 0.34	0.54 ± 0.25	t = 1.89 *P* = 0.06
**12 months**	0.47 ± 0.45	0.7 ± 0.16	t = 1.92 *P* = 0.06
**Paired t test**	t = 1.22 *P* = 0.23	t = 6.34 *P* < 0.001*	
**Palatal**			
**6 months**	0.53 ± 0.83	0.54 ± 0.42	t = 0.04 *P* = 0.96
**12 months**	0.62 ± 0.75	0.8 ± 0.4	t = 0.84 *P* = 0.4
**Paired t test**	t = 2.07 *P* = 0.055	t = 3.25 *P* = 0.005*	

SD: standard deviation, *P* ≤ 0.05 is statistically significant, analysis was done by independent t test. *: statistically significant

## Discussion

A growing number of patients have been requesting shorter treatment periods recently, which has led to the rise in popularity of immediate or early loading protocols, which are difficult to accomplish because of the inferior quality of bone in the posterior maxillary region [5]. Thus, the purpose of the current study was to compare the clinical and radiographic results of the undersized drilling technique versus the single drilling technique of an immediately loaded dental implant inserted in the posterior maxilla.

The survival rate of implants used in this study was 100%. This can be explained by the careful attention to factors like careful patient selection, patients’ cooperation and commitment to oral hygiene measures during the whole follow-up period, and the use of appropriate surgical techniques.

A successful dental implant depends on osseointegration, which is related to 6 main criteria: material biocompatibility, implant design, surface morphology, bone quality, surgical technique, and loading conditions [24]. Reducing surgical damage to bone tissue during the osteotomy is something that can be managed and could help to ensure the success of osseointegration. High temperatures during osteotomy preparation can modify tissue and kill cells. They can also have a detrimental impact on osseointegration, and cause crestal bone loss, and implant failure [16]. It has been suggested that the improvement of the drill design and technique to simplify the process and lower the possibility of the implant site overheating [10].

The undersized drilling technique is the reduction of the final diameter of the implant bone bed through an incomplete drilling sequence, which has been utilized to place implants in bone of poor quality [7]. Degidi et al. [25] claimed that the undersized drilling technique is an effective technique in low density bone.

The single drill system, while relatively newer in clinical practice, is a safe drilling protocol; it doesn’t produce more heat than the conventional drilling protocol. According to a previous study by Gehrke et al., [26] if the conventional gradual expansion method is simplified, implant bone apposition is still comparable to that of conventional techniques.

Insertion torque is the amount of force required to insert the implant into the osteotomy site [27]. As a measurement of primary stability, in our study, the mean insertion torque in the undersized drilling group was 35 ± 2.58 Ncm, while in the single drilling group, it was 35.62 ± 2.5 Ncm. Comparing both groups revealed no significant difference between them (*P* = 0.49). This result was in accordance with Benic et al., [28] who stated that the crucial criteria for immediate loading of implants is adequate insertion torque (ranging from 20 to 45 Ncm). Additionally, our study was consistent with Elian and Abdelaal [29], who recommended omitting the last drill for all preparations of the posterior maxillary implant sites to achieve high insertion torque. Also, this was in agreement with Senada et al., [17] who achieved insertion torque between 30 Ncm and 50 Ncm by using the single drilling technique. Our result was in line with Bisher and Khalil’s [15], who compared the single drilling technique with the conventional technique and obtained 35.08 ± 5.40 Ncm in the single drilling group compared to 27.18 ± 3.30 Ncm in the conventional group.

The undersized drilling technique had a mean ISQ value of 77.31 ± 3.92 at the time of implant insertion, 74.37 ± 3.2 after 3 months, 75.81 ± 4.06 after 6 months, and 76.12 ± 5.92 after 12 months, whereas the single drilling technique had a mean ISQ value of 76.25 ± 5.76 at the time of implant insertion, 73.18 ± 3.1 after 3 months, 74.93 ± 4.07 after 6 months, and 75.5 ± 4.53 after 12 months. Comparing both groups, there were no statistically significant differences regarding implant stability at different time intervals (*P* > 0.05).

Our study was in line with Elsheikh et al. [18], who claimed that the undersized drilling technique, when utilized in the posterior maxilla, exhibited better primary stability than other drilling techniques. Also, consistent with our results, Senada et al. [17] showed that the mean stability of the single drilling technique was 72.15 ± 9.33 after implant insertion. Our result was similar to the one obtained by Ahmed et al., [30] who had higher primary implant stability in the single drilling group than in the conventional group.

By compressing the bone, the undersized drilling technique results in increased bone-to-implant contact, leading to higher primary stability and insertion torque [31]. The undersized drilling technique may have a biological limit because according to Tabassum et al. [32], increased bone compression results in a poor tissue response during the early stages of healing.

Because only one drill was used, the single drilling technique showed high insertion torque and ISQ values. This can be attributed to the design of the drill [15]. Also, decreasing site preparation time, thus lessening bone tissue damage, may play a role in increasing the primary stability and insertion torque [33].

The increase in the bleeding during probing around a dental implant may give an indication of mucositis, which is considered an early marker of peri-implantitis [34]. After 3, 6, and 12 months of follow-up, there were no statistically significant differences between the two groups (*P* > 0.05). The controlled bleeding index of the patients was linked to the maintenance of oral hygiene measures during the follow-up period of this study. Our study was consistent with Sanz-Martín et al. [35] who claimed that oral hygiene measures and lower plaque accumulation affect modified bleeding scores directly, which are responsible for gingival inflammation.

When assessing the periodontium status, no significant differences were found between the two groups during the follow-up periods regarding peri-implant probing depth (*P* > 0.05). Peri-implant probing depth decreased from the time of crown loading till 12 months later in both groups. The values of peri-implant probing depth in both groups presented within the acceptable range of ≤ 4 mm. Our study was consistent with those of Kumar et al. [36], who observed that after 18 months of loading, the peri-implant probing depth decreased. Also, our result was in line with Ahmed et al., [30] who found no statistically significant difference between the single drilling group and the conventional one regarding the pocket depth after 6 months.

In terms of bone density, preoperative buccal and palatal bone density did not significantly differ between the two groups (*P* > 0.05), indicating that the two groups were homogeneous at the beginning of the study. Throughout the study, there was no significant difference between both groups in all intervals of assessment. By interpreting the results, the pairwise intragroup comparison showed a significant increase in buccal and palatal bone density after 12 months of loading when compared to preoperative values (*P* < 0.05), which indicates that the undersized drilling technique and the single drilling technique can be used successfully in increasing bone density in the posterior maxilla.

Our study was consistent with Herrero-Climent et al. [37] who found that the undersized drilling technique plays a role in improving bone density and increasing ISQ value. Meanwhile, Ahmed et al. [30] stated that using the single drilling approach in type IV bone would improve bone density. Additionally, Senada et al. [17] showed that the single drilling technique increases bone density significantly at 3 and 6-months intervals after implant insertion. Our results also supported a study done by Trisi et al. [38] who found that using a single drill to prepare the implant site greatly enhanced the peri-implant bone density in comparison to the initial host bone density. As opposed to our study, Hasan et al. [39] found that different patient responses resulted in variable bone responses after the immediate loading of the dental implants. After 6 months, 30% of patients had increased bone density, and 70% had decreased bone density.

Both groups had minimal buccal and palatal MBL, and after a year, there was no significant difference between them (*P* > 0.05). Our study was matched with the study of Elsheikh et al. [18] as they stated lack of statistically significant difference regarding MBL when comparing the undersized drilling technique with osseodensification and expanders. Also, Senada et al. [17] had similar results after using the single drilling technique. Our study was similar to the study made by Bisher and Khalil [15], who had 0.65 mm MBL after using the single drilling protocol.

Previous studies showed that MBL of 1.5 to 2 mm during the first year of functional loading associated with annual 0.2 mm bone loss was considered acceptable [40]. Our results indicate that both groups had less MBL than these studies during 12 months of functional loading.

### Limitations

This study was limited due to a small sample size and a short follow-up period. Stronger and more reliable results would come from a larger sample size and a longer follow-up time.

## Conclusion

Within the limits of this trial, both techniques revealed comparable and promising outcomes, but preparing the implant bed using the single drilling technique offers several merits for both the patient and clinician. In addition to avoiding excessive heat generation, mechanical damage, and high frictional forces during drilling procedures, surgical operations, and surgical site exposure take less time. Also, the single drill’s hollow design enables the collection of a bone core, which can be utilized in the self-bone grafting technique. Still, it requires a precise drilling orientation, as there are fewer opportunities for angulation corrections.

## Data Availability

The corresponding author can provide the data sets utilized and/or analyzed for this study upon reasonable request.

## Abbreviations

CBCT: Cone beam Computed tomography
HU: Hounsfield units
IRB: Institutional review board
ISQ: Implant stability quotient
mBI: Modified sulcus bleeding index
MBL: Marginal bone loss
RFA: Resonance frequency analysis
